# Supplementary material for: SIRT6 haploinsufficiency induces BRAFV600E melanoma cell resistance to MAPK inhibitors via IGF signalling
Source: Nat Commun. 2018 Aug 24;9:3440. doi: 10.1038/s41467-018-05966-z (PMC6109055; doi:10.1038/s41467-018-05966-z)
Supplement: Supplementary file 1 — Supplementary Information [file 41467_2018_5966_MOESM1_ESM.pdf]

SIRT6 haploinsufficiency induces BRAF^{V600E} melanoma cell resistance to MAPK inhibitors via IGF signalling

Strub et al.

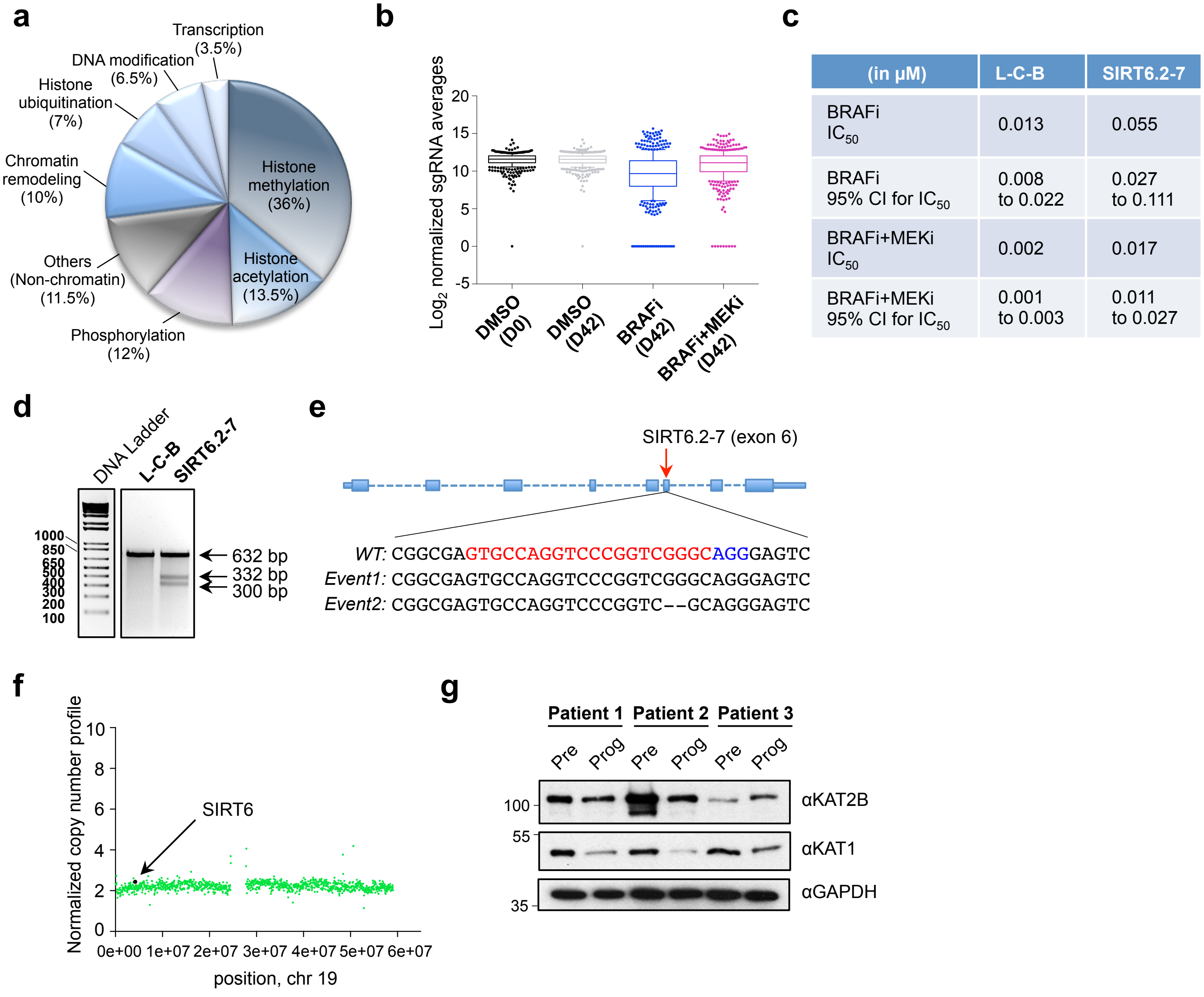

(in µM)	L-C-B	SIRT6.2-7
BRAFi IC_{50}	0.013	0.055
BRAFi 95% CI for IC_{50}	0.008 to 0.022	0.027 to 0.111
BRAFi+MEKi IC_{50}	0.002	0.017
BRAFi+MEKi 95% CI for IC_{50}	0.001 to 0.003	0.011 to 0.027

Supplementary Figure 1. Analysis of SIRT6-edited clones and additional screen validations. (**a**) Pie chart showing the composition of the chromatin CRISPR library used for the melanoma drug resistance screen. Numbers indicate the percentage of genes per category (total = 141 genes). (**b**) Box plot of the distribution of sgRNA read count averages (Log_2) for the indicated time points and treatments for each sgRNA. Bars represent 10-90 percentile. (**c**) IC_{50} and 95% CI (Confidence Interval) for IC_{50} are shown for L-C-B and SIRT6.2-7 cells in the indicated treatment. (**d**) SIRT6 gene was targeted using CRISPR-Cas9 generating the SIRT6.2-7 cell line. Efficiency of the sgRNA used was examined via SURVEYOR assay (from IDT Integrated DNA Technologies). Arrows indicate the non-cleaved or cleaved products of the samples indicated. (**e**) (Top) Region of the SIRT6 gene targeted, exons/coding regions are represented by blue boxes. (Bottom) CRISPR-Cas9 target sequence shown in red (PAM sequence in blue). Each edited allele is represented as event 1 and 2. (**f**) Control-FREEC[1] analyses of chromosome 19 in SKMel-239. Each green dot represents 50kb of the chromosome 19, no copy number gain or loss was detected for SIRT6 locus. (**g**) Immunoblot of KAT2B and KAT1 in the indicated whole cell lysates of paired patient-derived short-term cultures (prior to treatment (Pre) and upon drug resistance to vemurafenib (Prog)). GAPDH was used as loading control.

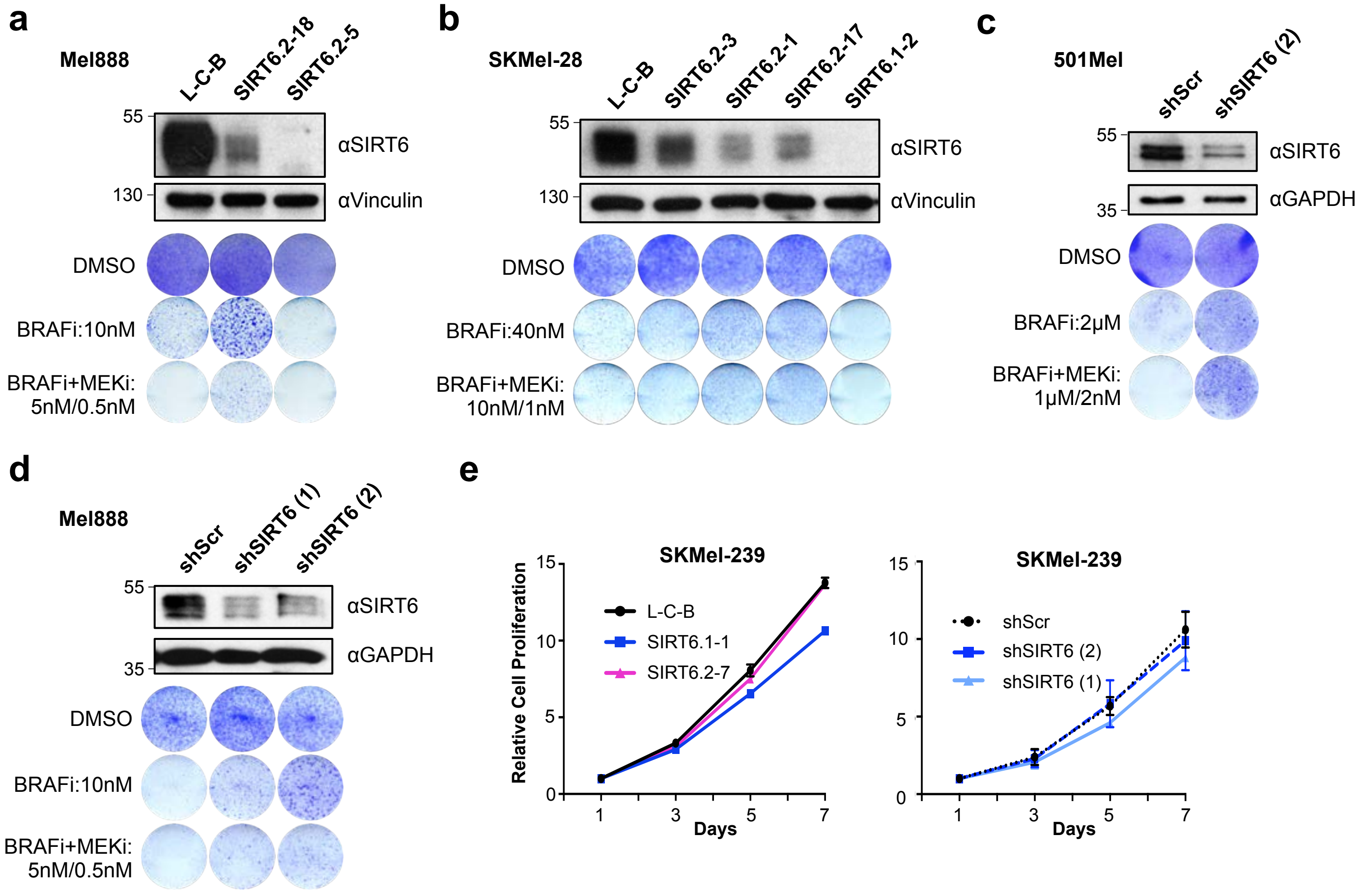

Supplementary Figure 2. SIRT6 knockdown decreases sensitivity to MAPKi in multiple BRAF^{V600E} melanoma cell lines. (**a, b**) SIRT6 immunoblot in the indicated whole cell lysates of Mel888 (a) and SKMel-28 (b) clonal CRISPR cell lines. Vinculin was used as a loading control. Cells were seeded at the same density and cultured in DMSO (1 week) or in the presence of the MAPKi (2 weeks) as indicated. (**c, d**) SIRT6 immunoblot in the indicated whole cell lysates from 501Mel (c) and Mel888 (d) cells. GAPDH used as loading control. Cells were seeded at the same density and cultured in DMSO (1 week) or in the presence of MAPKi (2 weeks) as indicated. **(e)** Proliferation curves of L-C-B, SIRT6.1-1 and SIRT6.2-7 cells (left) or SKMel-239 (right) expressing control or shRNAs against SIRT6, n=3. Data are mean ± SEM.

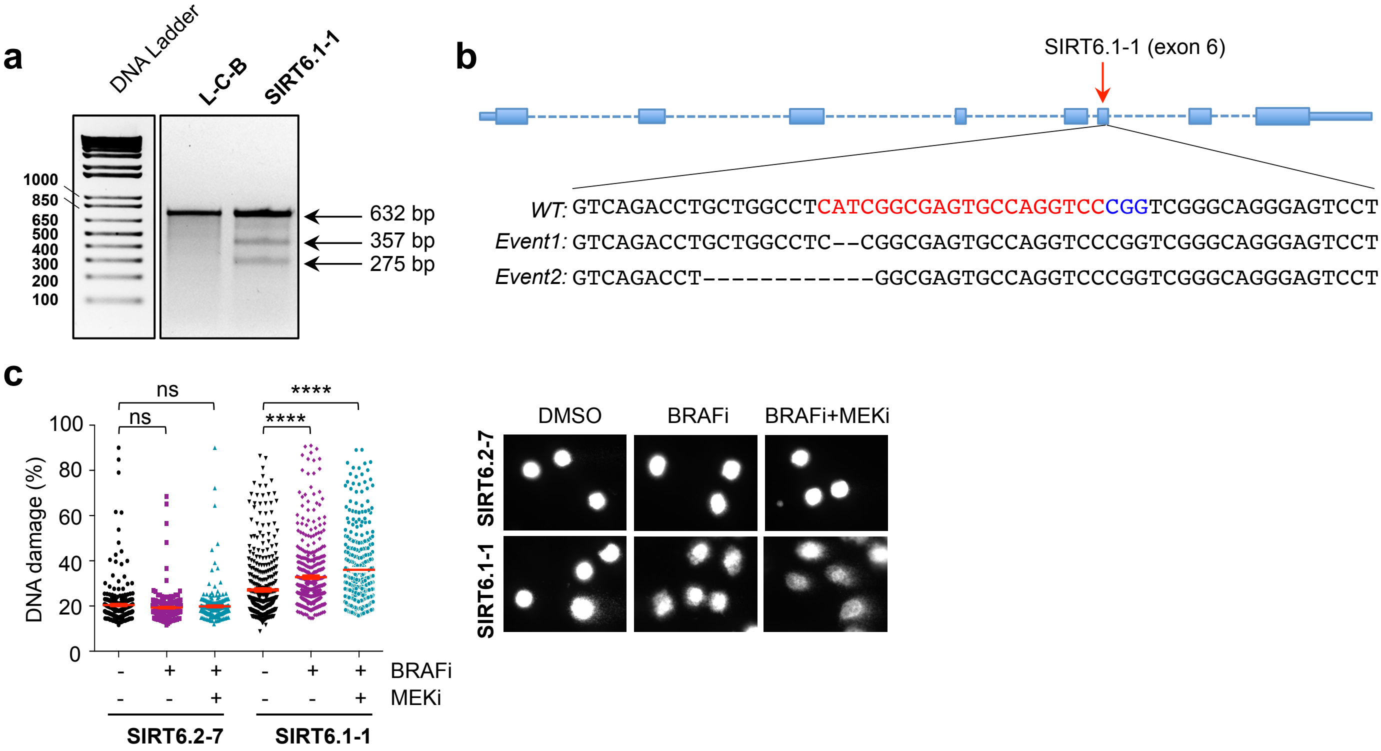

Supplementary Figure 3. SIRT6 bi-allelic edited clone shows elevated DNA damage. (**a**) SIRT6 gene was targeted using CRISPR-Cas9 generating the SIRT6.1-1 cell line. Efficiency of the sgRNA used was examined via SURVEYOR assay (IDT). Arrows indicate the non-cleaved or cleaved products. (**b**) Region of the SIRT6 gene targeted, exons/coding regions are represented by blue boxes. CRISPR-Cas9 target sequence is given in red (PAM sequence in blue). Each edited allele is represented as event 1 and 2. (**c**) (Left) Quantification of 3 independent comet assays (>100 cells analyzed per replicate). Data are mean ± SEM; ****P < 0.0001; One-way ANOVA used for comparisons. (Right) Representative slides of comet assay performed in SIRT6.2-7 and SIRT6.1-1 cells in the indicated conditions.

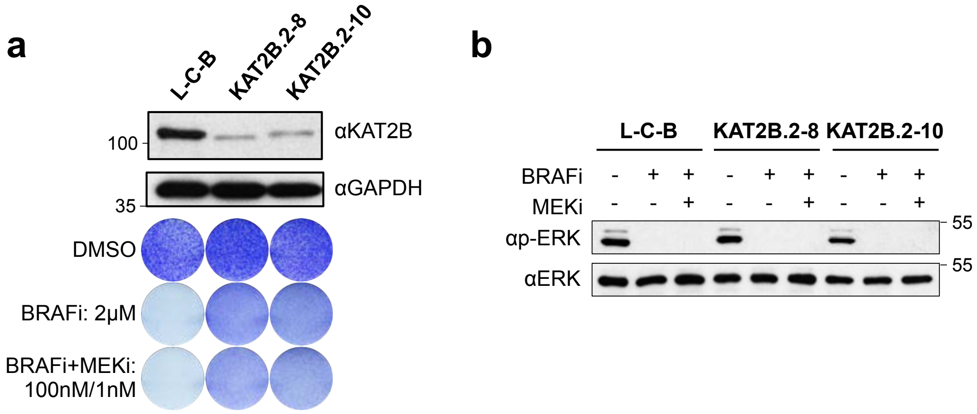

Supplementary Figure 4. KAT2B deficiency decreases sensitivity to MAPKi in an ERK-independent manner. (**a**) (Top) Immunoblot of KAT2B in the indicated CRISPR whole cell lysate samples. GAPDH used as loading control. (Bottom) Cells were seeded at the same density and cultured in DMSO (1 week) or in the presence of the MAPKi (2 weeks) as indicated. (**b**) Immunoblot of 2μM BRAFi or 100nM BRAFi+1nM MEKi treatment for 4 days on components of the ERK signaling pathway in L-C-B, KAT2B. 2-8 and KAT2B.2-10 cells.

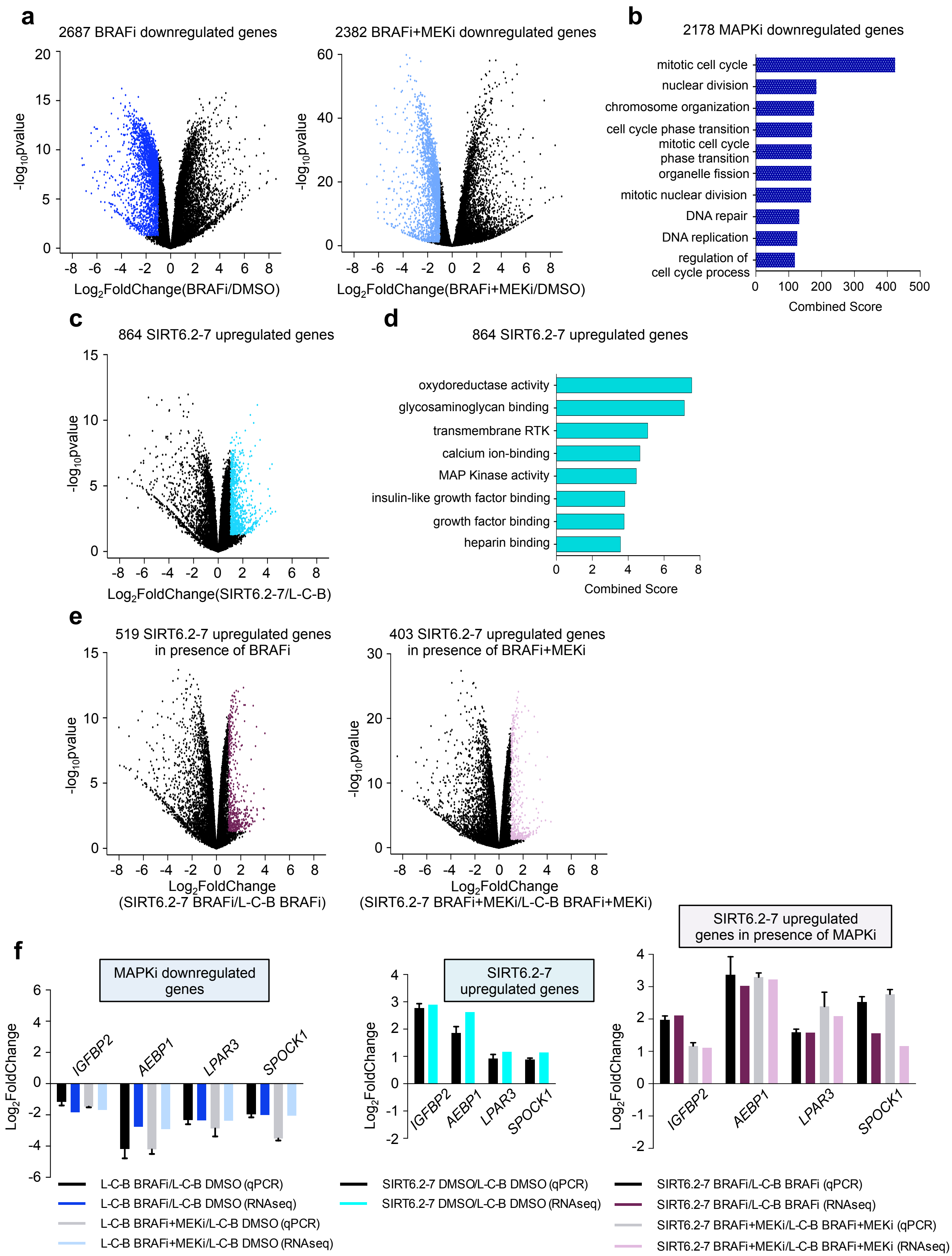

a
2687 BRAFi downregulated genes
-log10pvalue
Log2FoldChange(BRAFi/DMSO)
2382 BRAFi+MEKi downregulated genes
Log2FoldChange(BRAFi+MEKi/DMSO)
b
2178 MAPKi downregulated genes
mitotic cell cycle
nuclear division
chromosome organization
cell cycle phase transition
mitotic cell cycle phase transition
organelle fission
mitotic nuclear division
DNA repair
DNA replication
regulation of cell cycle process
Combined Score
c
864 SIRT6.2-7 upregulated genes
Log2FoldChange(SIRT6.2-7/L-C-B)
d
864 SIRT6.2-7 upregulated genes
oxydoreductase activity
glycosaminoglycan binding
transmembrane RTK
calcium ion-binding
MAP Kinase activity
insulin-like growth factor binding
growth factor binding
heparin binding
Combined Score
e
519 SIRT6.2-7 upregulated genes in presence of BRAFi
Log2FoldChange (SIRT6.2-7 BRAFi/L-C-B BRAFi)
403 SIRT6.2-7 upregulated genes in presence of BRAFi+MEKi
Log2FoldChange (SIRT6.2-7 BRAFi+MEKi/L-C-B BRAFi+MEKi)
f
MAPKi downregulated genes
SIRT6.2-7 upregulated genes
SIRT6.2-7 upregulated genes in presence of MAPKi
Log2FoldChange
IGFBP2
AEBP1
LPAR3
SPOCK1
L-C-B BRAFi/L-C-B DMSO (qPCR)
L-C-B BRAFi/L-C-B DMSO (RNAseq)
L-C-B BRAFi+MEKi/L-C-B DMSO (qPCR)
L-C-B BRAFi+MEKi/L-C-B DMSO (RNAseq)
SIRT6.2-7 DMSO/L-C-B DMSO (qPCR)
SIRT6.2-7 DMSO/L-C-B DMSO (RNAseq)
SIRT6.2-7 BRAFi/L-C-B BRAFi (qPCR)
SIRT6.2-7 BRAFi/L-C-B BRAFi (RNAseq)
SIRT6.2-7 BRAFi+MEKi/L-C-B BRAFi+MEKi (qPCR)
SIRT6.2-7 BRAFi+MEKi/L-C-B BRAFi+MEKi (RNAseq)

Supplementary Figure 5. Transcriptomic analyses of SIRT6 haploinsufficiency in the context of MAPKi. (**a**) Log_2 fold change of all SKMel-239 (L-C-B) genes upon four days of BRAFi or BRAFi+MEKi treatment over DMSO. Significantly downregulated genes shown in blue. (**b**) Functional annotation (biological processes) of the 2178 genes commonly downregulated after four days of BRAFi and BRAFi+MEKi treatment (MAPKi downregulated genes). Categories are ranked by combined score. (**c**) Log_2 fold change of all SIRT6.2-7 genes over L-C-B genes. Significantly upregulated genes are shown in light blue. (**d**) Functional annotation (molecular functions) of the 864 upregulated genes in the SIRT6.2-7 cells compared to L-C-B. Categories are ranked by combined score. (**e**) Log_2 fold change of all SIRT6.2-7 genes upon four days of BRAFi or BRAFi+MEKi treatment over L-C-B with MAPKi treatments. Significantly upregulated genes are shown in purple. (**f**) qRT-PCR analysis of genes identified in transcriptomic analyses (**Figure 3a**) in the indicated CRISPR cell lines and treatments, n=3. Data are mean ± SEM.

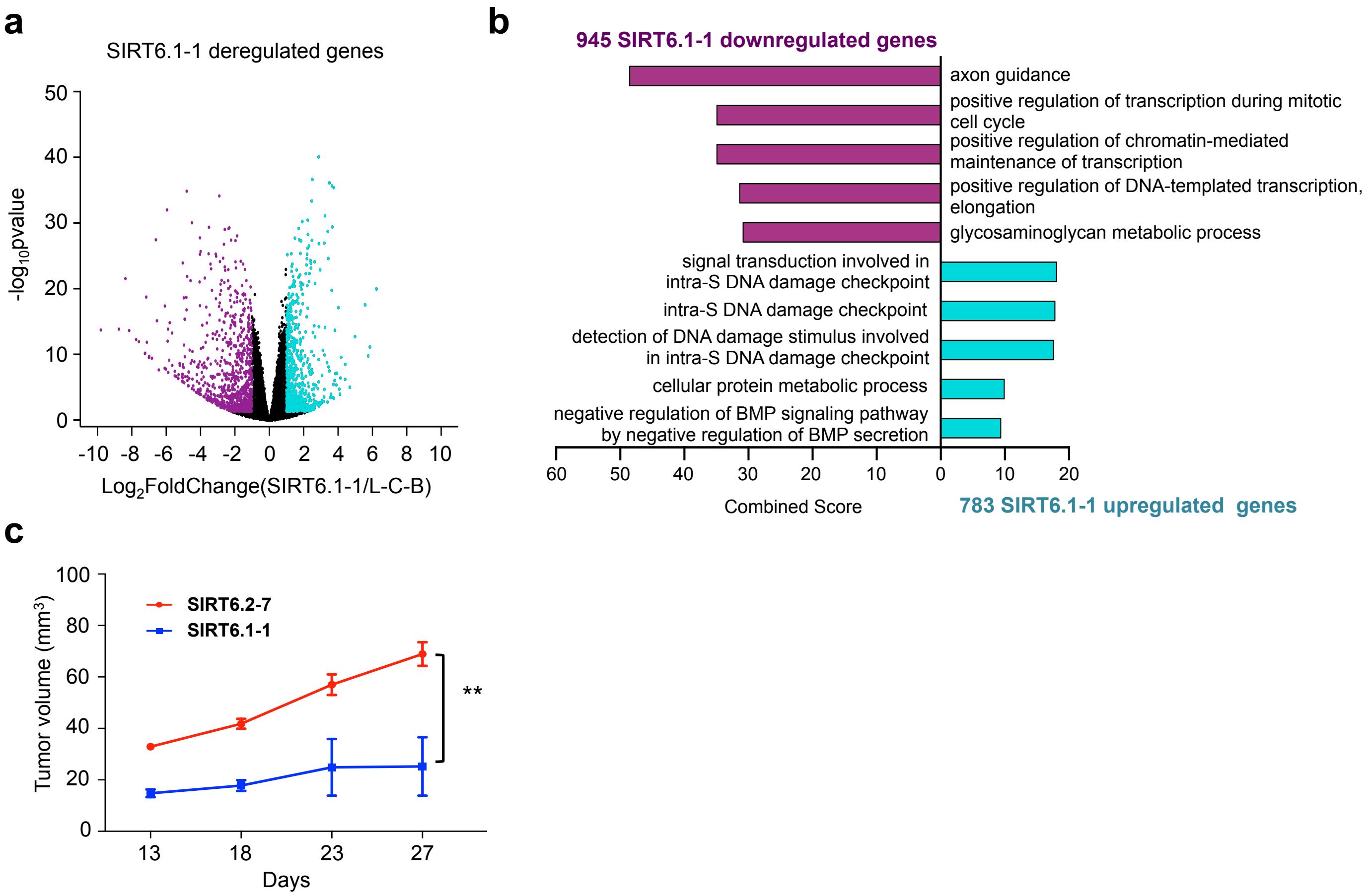

Supplementary Figure 6. Transcriptomic and functional analyses of bi-allelically edited clone SIRT6.1-1. (**a**) Log_2 fold change of all SIRT6.1-1 genes over L-C-B genes. Significantly downregulated genes are shown in purple and significantly upregulated genes are shown in light blue. (**b**) Functional annotation (biological processes) of the 945 downregulated genes in the SIRT6.1-1 cells compared to L-C-B and functional annotation (biological processes) of the 783 upregulated genes in the SIRT6.1-1 cells compared to L-C-B are shown. Categories are ranked by combined score. (**c**) Quantification of tumor volume in nude mice bearing xenograft tumors of SIRT6.2-7 or SIRT6.1-1 cells for 27 days. Mann-Whitney test was performed for comparison between the two groups of mice. Data are mean ± SEM. $^{**}P < 0.01$; n=5 for SIRT6.1-1 and n=28 for SIRT6.2-7.

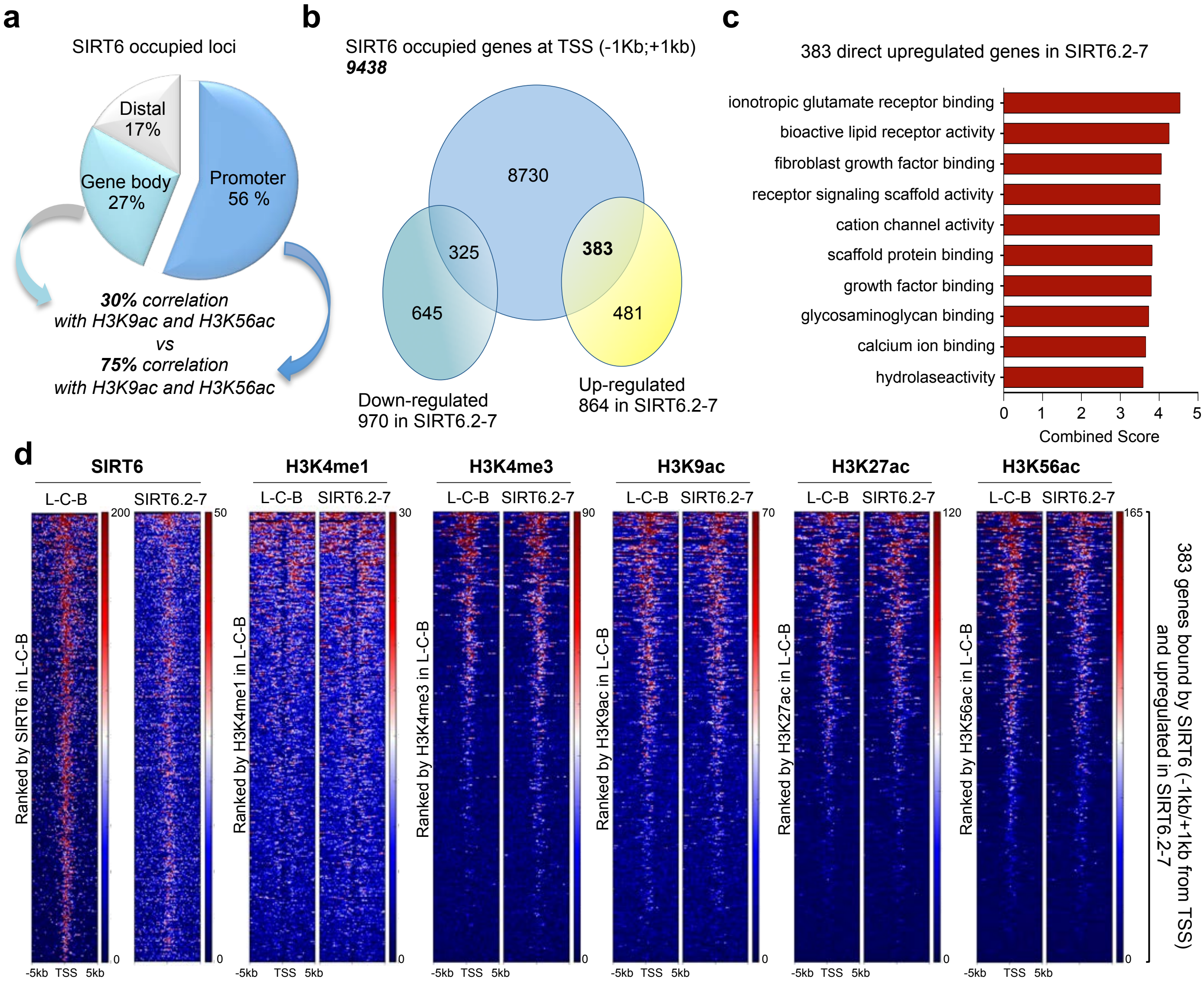

Supplementary Figure 7. SIRT6 target genes and associated histone modifications. (**a**) Pie chart representing distribution of SIRT6 peaks (in percentages) at promoters, gene bodies and distal regions. Promoters: -5 kb < TSS < 2 kb; gene bodies: 2 kb > TSS to TES; other regions are defined as distal. (TSS: transcription start site, TES: transcription end site). Correlation with H3K9ac and H3K56ac is shown (in percentages). (**b**) Venn diagram displaying SIRT6-occupied genes at TSS (-1kb < TSS > +1kb) and deregulated in SIRT6.2-7 cells. 325 and 383 genes are respectively downregulated or upregulated in SIRT6.2-7 cells and bound by SIRT6 in L-C-B cells. (**c**) Functional annotation (molecular functions) of the 383 upregulated genes in the SIRT6.2-7 cells vs. L-C-B cells and bound by SIRT6 in L-C-B cells. Categories are ranked by combined score*.* (**d**) Heatmaps for ChIP-seq reads for SIRT6, H3K4me1, H3K4me3, H3K9ac, H3K27ac and H3K56ac in L-C-B or SIRT6.2-7 cells, ranked from high to low for SIRT6 or histone modification in L-C-B cells. Data is centered on ±5kb window around the TSS of 383 SIRT6.2-7 upregulated genes and bound by SIRT6 in L-C-B cells. Enrichment scales shown on right.

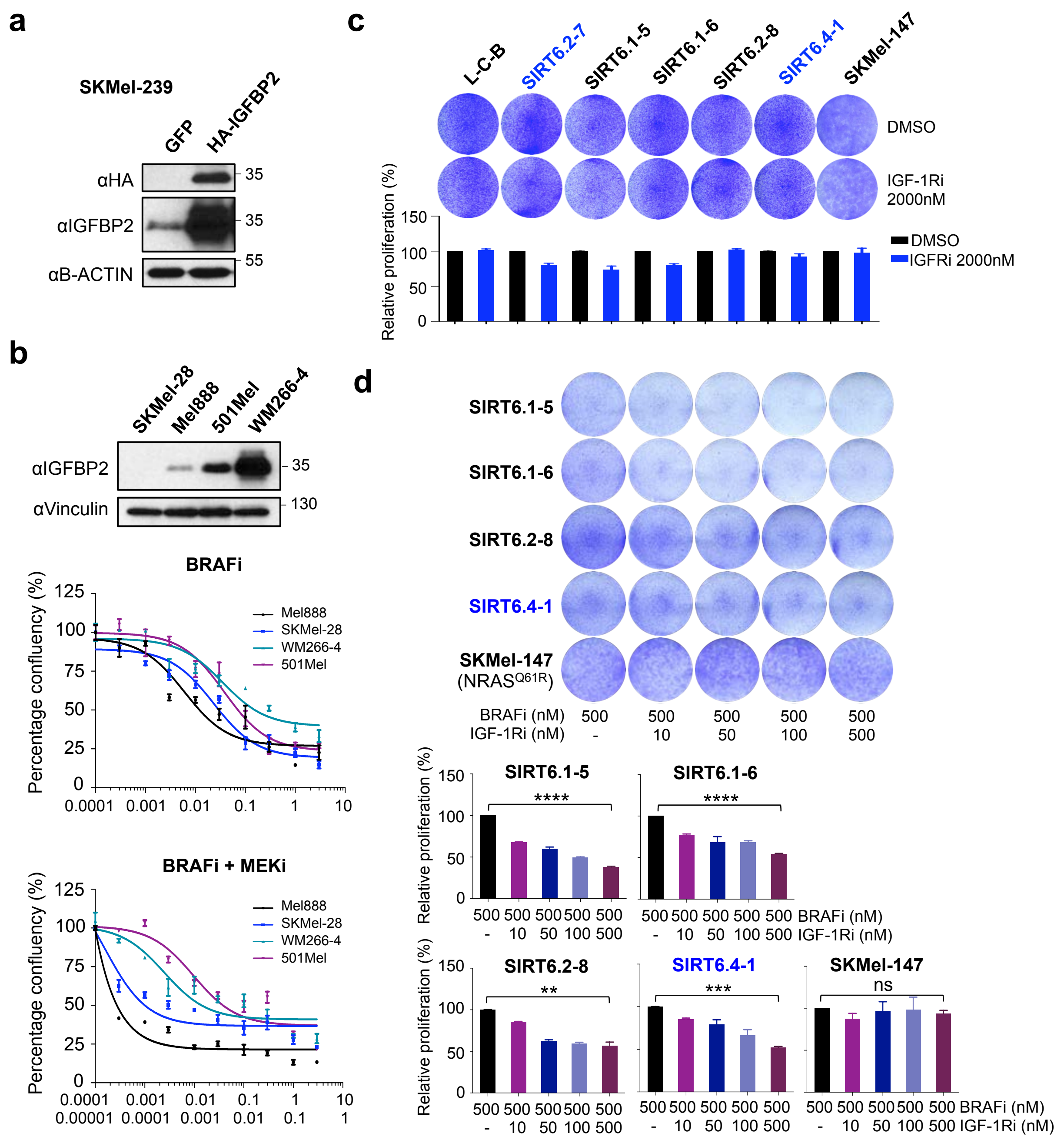

Supplementary Figure 8. Insulin signaling and IGFBP2 promote resistance to MAPKi. (**a**) Immunoblot of IGFBP2 or HA in the indicated whole cell lysates. B-Actin used as loading control. (**b**) (Top) Immunoblot of IGFBP2 in the indicated whole cell lysate samples. Vinculin used as loading control. (Bottom) Growth inhibition curves for BRAFi and BRAFi+MEKi in the indicated cell lines and conditions (μM) after 72h, n=3. Data are mean ± SEM. (**c**) (Top) Indicated clones were seeded at the same density and cultured for one week in presence of DMSO or IGF-1Ri. (Bottom) Relative cell proliferation (percentage) of the above, n=3. Data are mean ± SEM. (**d**) (Top) Indicated cell lines were seeded at the same density and cultured for one week in presence of the indicated compounds. (Bottom) Relative cell proliferation (percentage) of the same cells, n=3. Data are mean ± SEM; $^{**}P < 0.01$, $^{***}P < 0.001$, $^{****}P < 0.0001$; Paired two-tailed Student's *t*-test was performed for comparisons. Skmel-147 ($NRAS^{Q61R}$-mutant melanoma) used for linsitinib toxicity control.

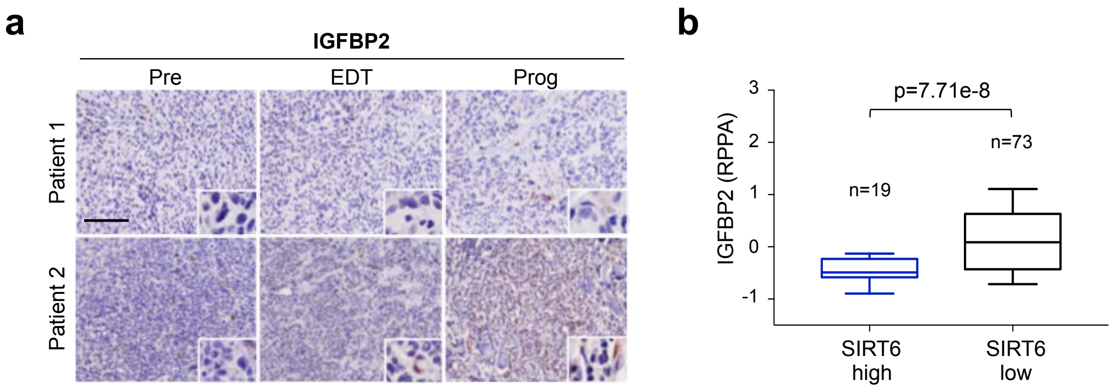

Supplementary Figure 9. SIRT6 and IGFBP2 levels anti-correlate in human melanoma samples. (**a**) IGFBP2 IHC in paired tumor samples (Patient 1 and 2, **Supplementary Table 5**) taken before treatment (Pre), early during treatment (EDT) and on disease progression (Prog). Images 10X; insets 40x. Scale bar represents 100μm. Scores are in main (**Fig. 5d**). **(b)** Box plot of IGFBP2 protein expression (RPPA) from TCGA samples (primary tumors) classified by SIRT6 alteration, high SIRT6 mRNA expression in blue (z-score threshold > 1) or low SIRT6 mRNA expression in black (z-score threshold < 1). Bars represent 10-90 percentile. p-Values derived from Student t-test.

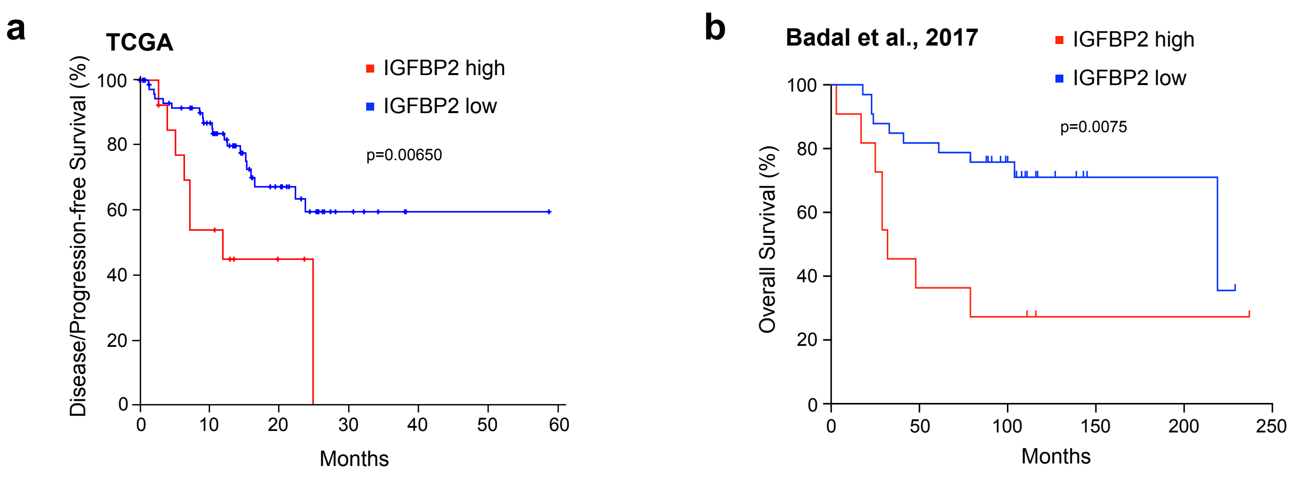

Supplementary Figure 10. Elevated IGFBP2 expression is associated with poor prognosis in melanoma patients. (**a**) Kaplan-Meier analysis for Disease/Progression-free survival stratified by IGFBP2 protein expression (RPPA) from TCGA samples (primary tumors, n=92). High IGFBP2 protein level in red; z-score threshold > 0.5 or low IGFBP2 protein level in blue; z-score threshold < 0.5. P-Values, log rank test. (**b**) Kaplan-Meier analysis for Overall Survival stratified by IGFBP2 expression (mRNA) from 44 primary tumors[2]. High IGFBP2 mRNA expression level in red (top quartile) or low IGFBP2 mRNA expression level in blue (first to third quartile). P-Values, log rank test.

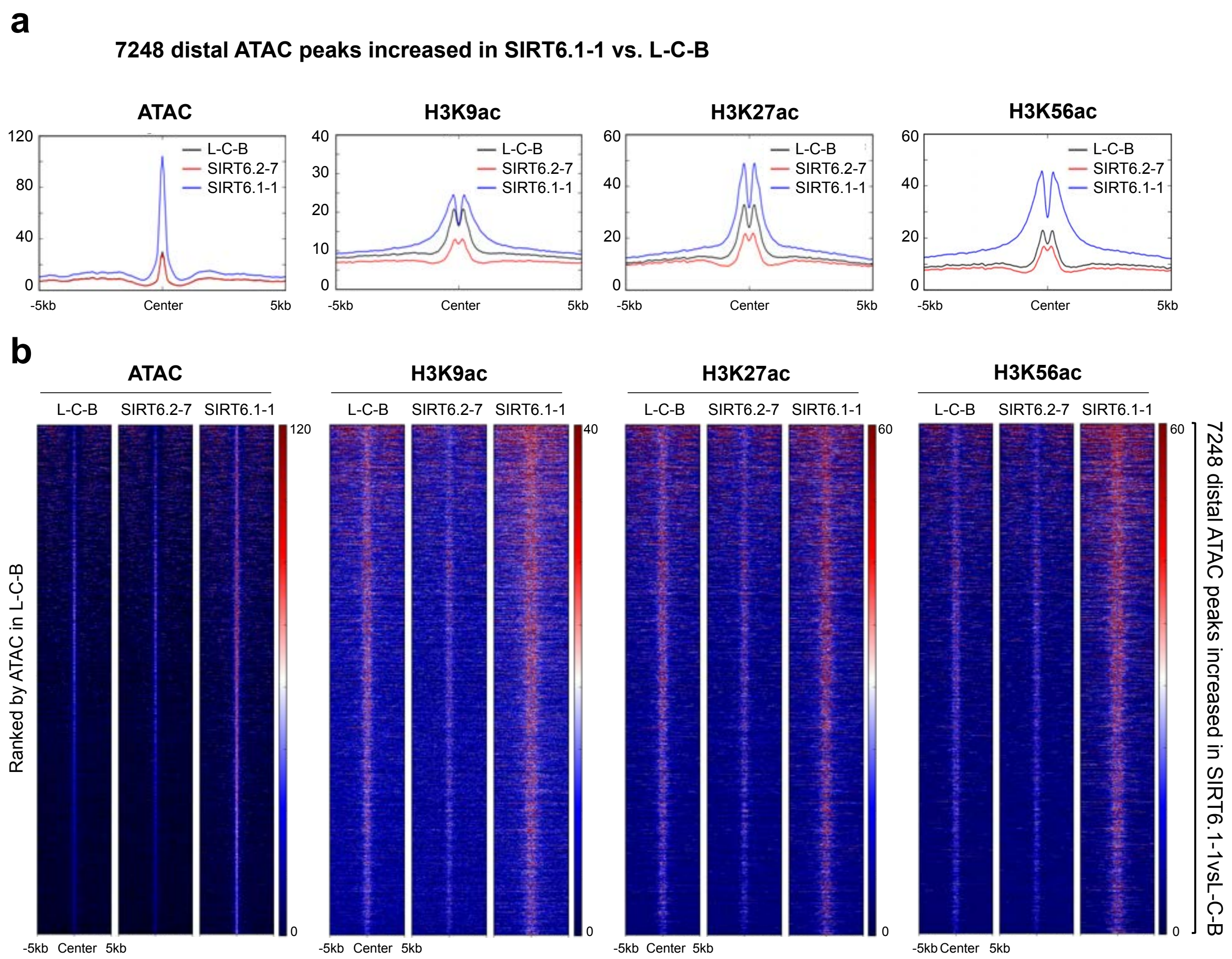

Supplementary Figure 11. SIRT6 bi-allelically edited clone gains chromatin accessibility and histone acetylation at distal regulatory regions. (**a**) ATAC-seq, H3K9ac, H3K27ac and H3K56ac ChIP-seq meta-profiles in L-C-B, SIRT6.2-7 and SIRT6.1-1 cells. Data is centered on ±5kb window around the center of 7248 distal ATAC peaks increased in SIRT6.1-1 cells compare to L-C-B cells (**Supplementary Table 4**). Distal peaks are defined as excluded from -1kb/+1kb around the TSS (TSS: transcription start site). Enrichment scales shown on the left. (**b**) Heatmaps for ATAC-seq and ChIP-seq reads for H3K9ac, H3K27ac and H3K56ac in L-C-B, SIRT6.2-7 and SIRT6.1-1 cells, ranked from high to low ATAC signal in L-C-B cells. Data is centered as in (**a**). Enrichment scales shown on the right.

Figure 1c

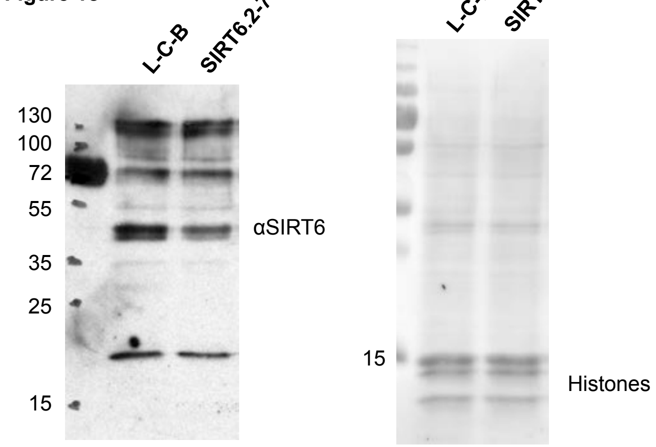

Figure 1e

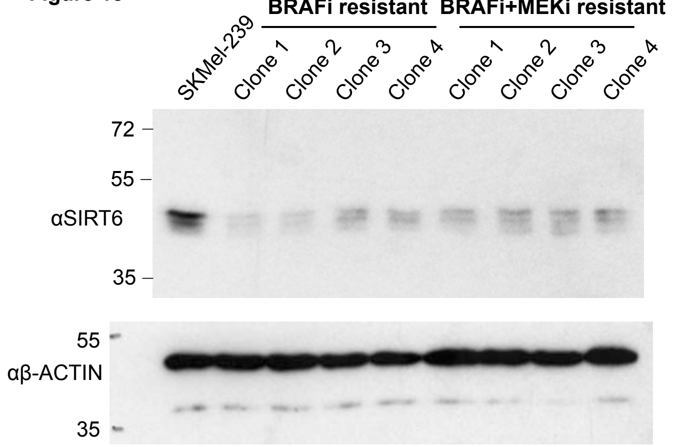

Figure 1f

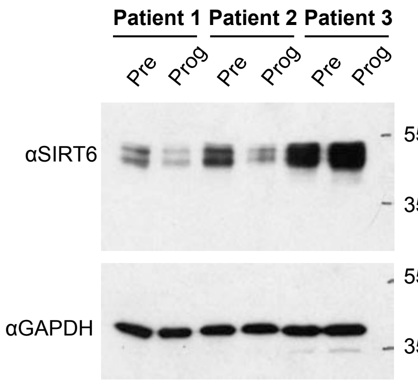

Figure 2a

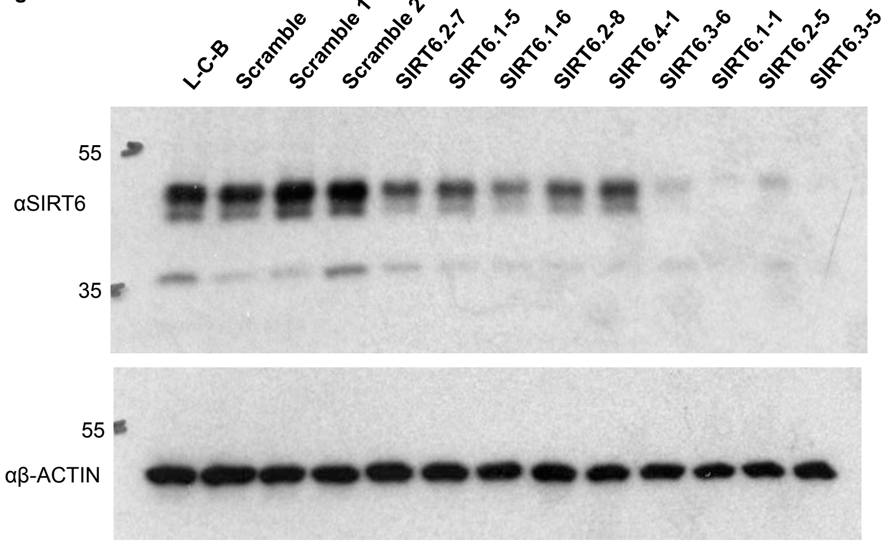

Figure 2b

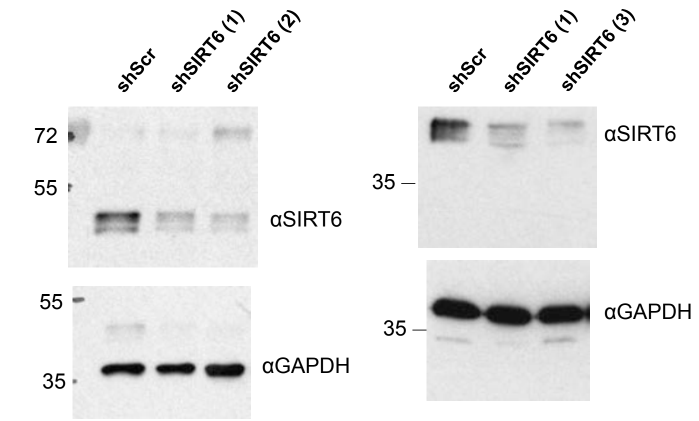

Figure 2d

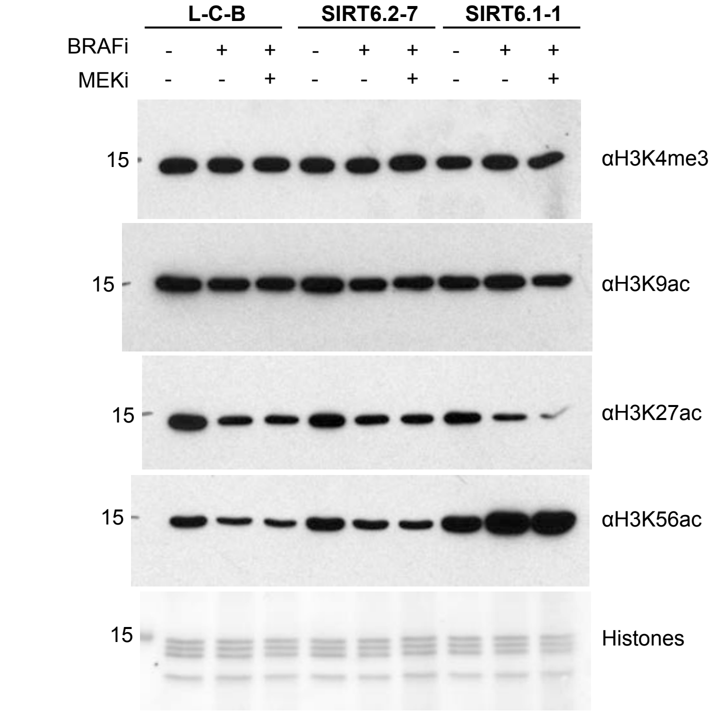

Figure 2e

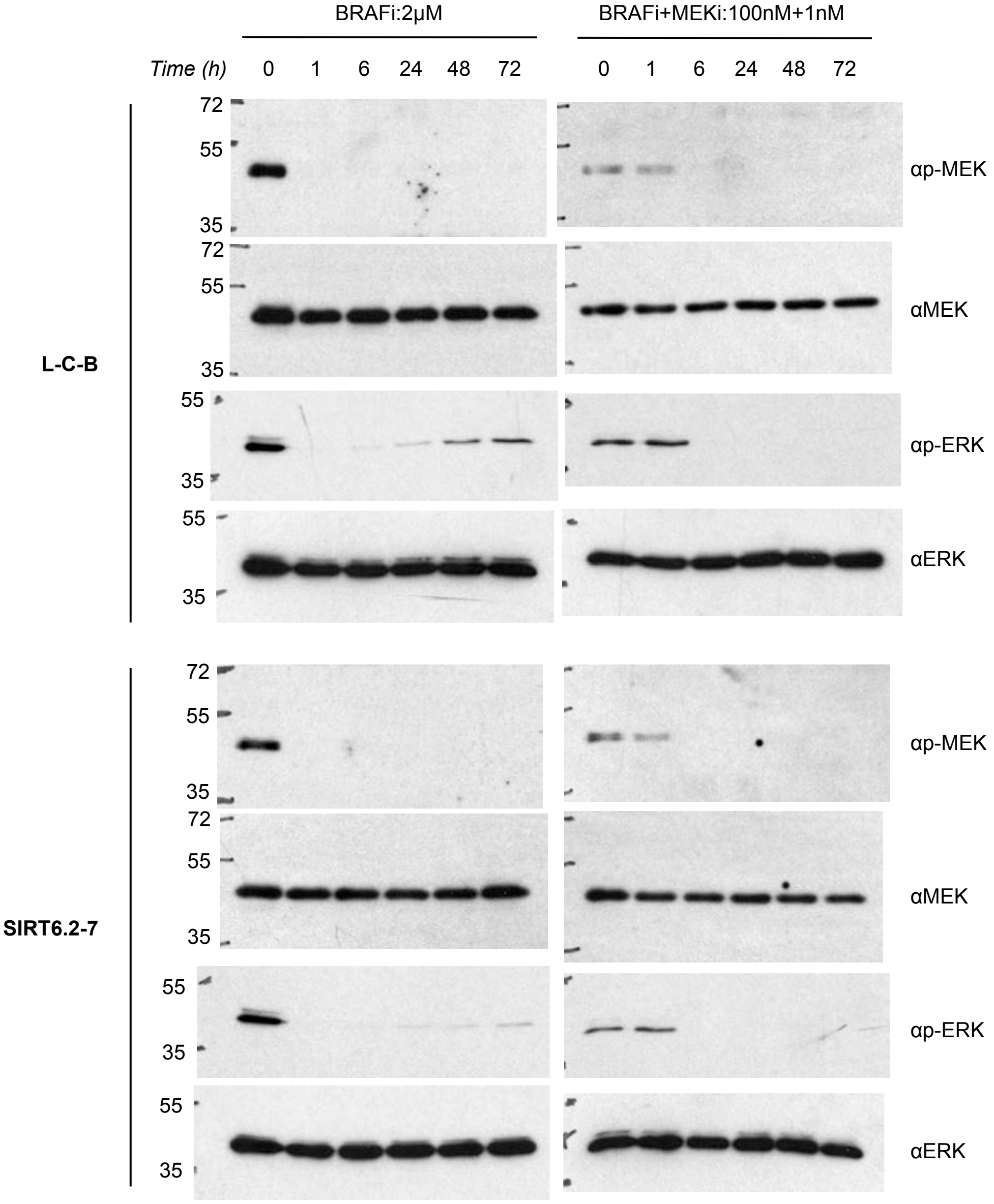

Figure 2f

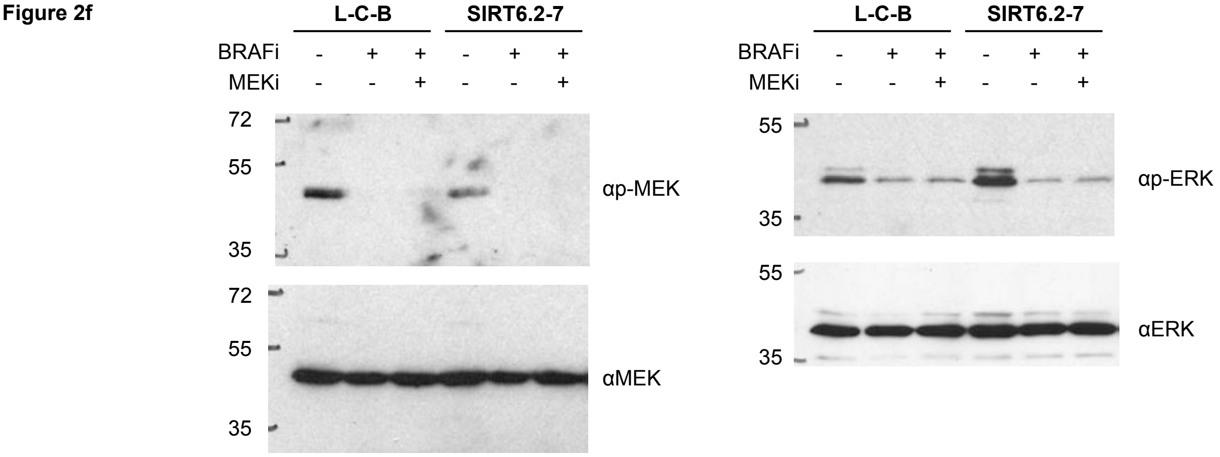

Figure 3b

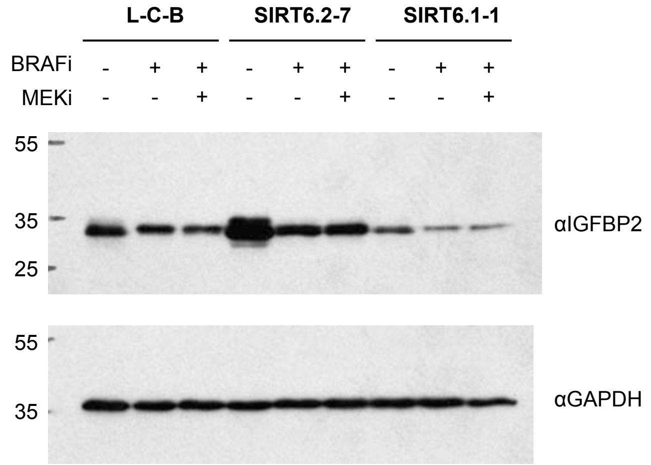

Figure 4b

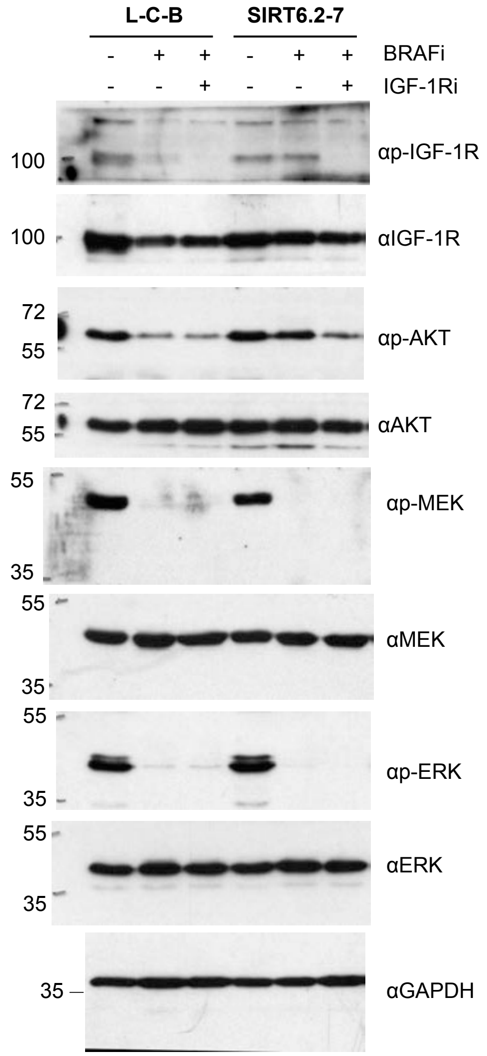

Supplementary Figure 12. Original immunoblot scans of key data points. Uncropped images of key immunoblots from main figures. Relevant figures are indicated on top and antibodies are referenced beside each immunoblot.

Supplementary References

1. Boeva, V. *et al.* Control-FREEC: a tool for assessing copy number and allelic content using next-generation sequencing data. *Bioinformatics* (2012). doi:10.1093/bioinformatics/btr670
2. Badal, B. *et al.* Transcriptional dissection of melanoma identifies a high-risk subtype underlying TP53 family genes and epigenome deregulation. *JCI insight* (2017). doi:10.1172/jci.insight.92102
