# Supplementary material for: SIRT6 haploinsufficiency induces BRAFV600E melanoma cell resistance to MAPK inhibitors via IGF signalling
Source: Nat Commun. 2018 Aug 24;9:3440. doi: 10.1038/s41467-018-05966-z (PMC6109055; doi:10.1038/s41467-018-05966-z)
Supplement: Supplementary file 3 — Description of Additional Supplementary Files [file 41467_2018_5966_MOESM3_ESM.pdf]

## Description of Additional Supplementary Files

**File Name: Supplementary Data 1**
**Description:** CRISPR-Cas9 screen to identify chromatin regulators of melanoma resistance to MAPKi. [Tables contain genes targeted with 3-4 sgRNAs and sgRNA sequences, normalized read counts for each conditions and their averages, Log2FoldChanges with (-)Log10 p values, as well as top hits represented for each condition.]

**File Name: Supplementary Data 2**
**Description:** Transcriptional profiling of MAPKi treatments in BRAFV600E melanoma cells. [Tables contain RNA-seq data showing altered genes under various conditions to identify potential targets involved in MAPKi resistance in SIRT6 haploinsufficient and KO cells.]

**File Name: Supplementary Data 3**
**Description:** RPPA data of L-C-B (control), SIRT6.2-7 or SIRT6.1-1 cells in the presence or absence of MAPKi. [Table contains RPPA of differential proteins and post-translational modifications present in the indicated conditions.]

**File Name: Supplementary Data 4**
**Description:** SIRT6 regulatory network and chromatin accessibility data. [Tables with ChIPseq data to identify SIRT6 direct targets and its role in regulating the chromatin environment.]

**File Name: Supplementary Data 5**
**Description:** Patient characteristics and IHC scores. [Tables with patient clinical information and IHC scores for SIRT6 and IGFBP2.]

**File Name: Supplementary Data 6**
**Description:** Primers used for qRT-PCR in this study.
